# Point-of-Care Ultrasound (POCUS) Literature Primer: Key Papers on Renal and Biliary POCUS

**DOI:** 10.7759/cureus.37294

**Published:** 2023-04-08

**Authors:** Daniel J Kim, Colin R Bell, Tomislav Jelic, Rajiv Thavanathan, Claire L Heslop, Frank Myslik, David Lewis, Paul Atkinson, Jordan Chenkin, Ian M Buchanan, Paul Olszynski, Gillian Sheppard, Talia Burwash-Brennan, Elizabeth Lalande

**Affiliations:** 1 Department of Emergency Medicine, University of British Columbia, Vancouver, CAN; 2 Department of Emergency Medicine, Vancouver General Hospital, Vancouver, CAN; 3 Department of Emergency Medicine, University of Calgary, Calgary, CAN; 4 Department of Emergency Medicine, University of Manitoba, Winnipeg, CAN; 5 Department of Emergency Medicine, University of Ottawa, Ottawa, CAN; 6 Department of Medicine, Division of Emergency Medicine, University of Toronto, Toronto, CAN; 7 Department of Emergency Medicine, Schulich School of Medicine and Dentistry, University of Western Ontario, London, CAN; 8 Department of Emergency Medicine, Saint John Regional Hospital, Saint John, CAN; 9 Department of Emergency Medicine, Dalhousie University New Brunswick, Saint John, CAN; 10 Department of Medicine, Division of Emergency Medicine, McMaster University, Hamilton, CAN; 11 Department of Emergency Medicine, University of Saskatchewan, Saskatoon, CAN; 12 Department of Emergency Medicine, Memorial University of Newfoundland, St. John's, CAN; 13 Department of Family and Emergency Medicine, Université de Montréal, Montreal, CAN; 14 Department of Emergency Medicine, Université Laval, Quebec City, CAN

**Keywords:** cholecystitis, cholelithiasis, biliary, renal colic, hydronephrosis, renal, ultrasound, point-of-care-ultrasound

## Abstract

Objective

The objective of this study is to identify the top five influential papers published on renal point-of-care ultrasound (POCUS) and the top five influential papers on biliary POCUS in adult patients.

Methods

A 14-member expert panel was recruited from the Canadian Association of Emergency Physicians (CAEP) Emergency Ultrasound Committee and the Canadian Ultrasound Fellowship Collaborative. All panel members have had ultrasound fellowship training or equivalent, are actively engaged in POCUS scholarship, and are involved with POCUS at their local site and nationally in Canada. We used a modified Delphi process consisting of three rounds of sequential surveys and discussion to achieve consensus on the top five influential papers for renal POCUS and biliary POCUS.

Results

The panel identified 27 relevant papers on renal POCUS and 30 relevant papers on biliary POCUS. All panel members participated in all three rounds of the modified Delphi process, and after completing this process, we identified the five most influential papers on renal POCUS and the five most influential papers on biliary POCUS.

Conclusion

We have developed a list, based on expert opinion, of the top five influential papers on renal and biliary POCUS to better inform all trainees and clinicians on how to use these applications in a more evidence-based manner. This list will also be of interest to clinicians and researchers who strive to further advance the field of POCUS.

## Introduction

Point-of-care ultrasound (POCUS) has been adopted by a wide range of specialties, including critical care, internal medicine, anesthesia, hospital medicine, and pediatrics [1]. Notably, emergency medicine (EM) was a pioneer for being one of the earliest specialties to endorse the use of POCUS at a broad national organizational level: the American College of Emergency Physicians (ACEP) first endorsed its use in 1990 [2] and the Canadian Association of Emergency Physicians (CAEP) in 1999 [3]. The specialty of EM has also been trailblazing in its work on POCUS education [4], clinical guideline creation [2,3], administrative integration [5], and research [6,7]. The POCUS literature base continues to grow at a dramatic rate [6,7]. Although many important POCUS papers have been published, there are very few publications that have systematically identified the most influential papers in this field [8].

The objective of this series is to systematically generate a list of papers for each major application or use of POCUS. The first publication in this series identified the top five influential papers published on focused assessment with sonography in trauma (FAST) and extended FAST (E-FAST) [8]. Such a resource is useful to educate residents, fellows, and engaged physicians on the literature base supporting their use of POCUS as well as to inform clinicians in all specialties on how to use POCUS in an evidence-based manner. It can also be an effective resource to summarize the POCUS literature to help researchers develop impactful future studies. The objective of this study is to use a modified Delphi process to identify the five most influential papers published on renal POCUS and the five most influential papers published on renal and biliary POCUS in adult patients.

## Materials and methods

Study design

This was a modified Delphi process [9,10] using sequential surveys and discussion amongst the expert panel to build consensus and identify the most influential papers on renal and biliary POCUS. This is part of an ongoing series to identify the most influential papers for each major application or use of POCUS. We applied the same study design and protocol we previously described in the initial paper of this series [8].

Participants

We recruited a 14-member expert panel from the Canadian Association of Emergency Physicians (CAEP) Emergency Ultrasound Committee and the Canadian Ultrasound Fellowship Collaborative (Appendix 1). All panel members have had ultrasound fellowship training or equivalent, are actively engaged in POCUS scholarship, and are involved with POCUS at their local site and nationally in Canada. We invited these individuals by email to participate in this modified Delphi process.

Modified Delphi process

Our modified Delphi process involved three rounds of surveys using Qualtrics (Qualtrics International, Seattle, WA) distributed by email in three-week intervals starting in October 2022. Although individual submissions were anonymized, the results of each round were distributed to all members of the panel.

We described our modified Delphi process in the initial publication of this series [8]. To briefly summarize, in round one, participants nominated five to 10 papers they considered the most influential for renal POCUS and five to 10 papers for biliary POCUS. We defined the term "influential" as important in informing practitioners on how to use POCUS in clinical practice. These papers were then collated into the survey instrument for round two, where participants were asked to select their five most influential papers for both renal and biliary POCUS. Papers receiving two or fewer votes were excluded, but panel members were allowed to advocate for the inclusion of a maximum of one of these papers per panel member for inclusion in the third round. The expert panel met virtually on Zoom (Zoom Video Communications Inc., San Jose, CA) on November 29, 2022, to discuss the results of round two. In the third round of the remaining papers, participants were asked to rank their top five papers for renal POCUS and biliary POCUS in descending order. The most influential paper was given a score of five, the next most influential was given a score of four, and so forth until the least influential paper was given a score of one. At the end of round three, these scores were added together, with the five highest-scoring papers identified as the most influential papers for renal and biliary POCUS.

There were no exclusions based on the publication date, type of study, or language of the paper. However, any studies enrolling only pediatric patients were excluded.

## Results

All 14 members of the expert panel participated in all three rounds of the modified Delphi process. The appendix lists the members of the panel and their academic affiliations. After round one, a total of 28 nominations were provided for renal POCUS, but one nomination was excluded as it was not a published paper; therefore, 27 papers were included in round two. For biliary POCUS, 30 papers were nominated and included in round two. After round two, there were nine candidate papers for renal POCUS and 12 candidate papers for biliary POCUS in round three. The three-round voting process generated a rank-order list of these papers in order of most to least influential in Table 1 and Table 2. The study populations of the top five papers for renal and biliary POCUS included only ED patients.

**Table 1:** All renal POCUS papers eligible for round three of the Delphi process, along with their votes in round two and their votes and total score in round three. The top five papers are indicated in the "final rank" column.

Paper	Round two votes for the top five (No (%))	Round three votes for the top five (No (%))	Round three total score	Final rank
Smith-Bindman R et al.: Ultrasonography versus computed tomography for suspected nephrolithiasis. N Engl J Med. 2014;371(12):1100–10. [11]	9 (64%)	13 (93%)	59	1
Wong C et al.: The accuracy and prognostic value of point-of-care ultrasound for nephrolithiasis in the emergency department: a systematic review and meta-analysis. Acad Emerg Med. 2018;25(6):684–98. [12]	8 (57%)	12 (86%)	43	2
Gaspari RJ, Horst K.: Emergency ultrasound and urinalysis in the evaluation of flank pain. Acad Emerg Med, 2005;12(12):1180–4. [13]	6 (43%)	12 (86%)	38	3
Wang RC et al. Effect of an ultrasound-first clinical decision tool in emergency department patients with suspected nephrolithiasis: a randomized trial. Am J Emerg Med. 2022;60:164–70. [14]	5 (36%)	8 (57%)	18	4
Taylor M et al.: Ultrasonography for the prediction of urological surgical intervention in patients with renal colic. Emerg Med J. 2016;33(2):118–23. [15]	5 (36%)	7 (50%)	17	5
Herbst MK et al.: Effect of provider experience on clinician-performed ultrasonography for hydronephrosis in patients with suspected renal colic. Ann Emerg Med. 2014;64(3):269–76. [16]	4 (29%)	7 (50%)	15	
Daniels B et al.: STONE PLUS: evaluation of emergency department patients with suspected renal colic, using a clinical prediction tool combined with point-of-care limited ultrasonography. Ann Emerg Med. 2016;67(4):439–48. [17]	6 (43%)	6 (43%)	12	
Goertz JK, Lotterman S.: Can the degree of hydronephrosis on ultrasound predict kidney stone size? Am J Emerg Med. 2010;28(7):813–6. [18]	3 (21%)	3 (21%)	6	
Pathan SA et al.: Emergency physician interpretation of point-of-care ultrasound for identifying and grading of hydronephrosis in renal colic compared with consensus interpretation by emergency radiologists. Acad Emerg Med. 2018;25(10):1129–37. [19]	3 (21%)	2 (14%)	2	

**Table 2:** All biliary POCUS papers eligible for round three of the Delphi process, along with their votes in round two and their votes and total score in round three. The top five papers are indicated in the "final rank" column.

Paper	Round two votes for the top five (No (%))	Round three votes for the top five (No (%))	Round three total score	Final rank
Ross M et al.: Emergency physician-performed ultrasound to diagnose cholelithiasis: a systematic review. Acad Emerg Med. 2011;18(3):227–35. [20]	8 (57%)	10 (71%)	42	1
Hilsden R et al.: Point of care biliary ultrasound in the emergency department (BUSED) predicts final surgical management decisions. Trauma Surg Acute Care Open. 2022;7(1):e000944. [21]	6 (43%)	9 (64%)	28	2
Summers SM et al.: A prospective evaluation of emergency department bedside ultrasonography for the detection of acute cholecystitis. Ann Emerg Med. 2010;56(2):114–22. [22]	6 (43%)	8 (57%)	26	3
Blaivas M et al.: Decreasing length of stay with emergency ultrasound examination of the gallbladder. Acad Emerg Med. 1999;6(10):1020–3. [23]	5 (36%)	7 (50%)	24	4
Kendall JL, Shimp RJ.: Performance and interpretation of focused right upper quadrant ultrasound by emergency physicians. J Emerg Med. 2001;21(1):7–13. [24]	4 (29%)	5 (36%)	21	5
Gaspari RJ et al.: Learning curve of bedside ultrasound of the gallbladder. J Emerg Med. 2009;37(1):51–6. [25]	5 (36%)	9 (64%)	20	
Sharif S et al.: Evaluating the diagnostic accuracy of point-of-care ultrasound for cholelithiasis and cholecystitis in a Canadian emergency department. CJEM. 2021;23(5):626–30. [26]	6 (43%)	7 (50%)	17	
Hilsden R et al.: Point-of-care biliary ultrasound in the emergency department (BUSED): implications for surgical referral and emergency department wait times. Trauma Surg Acute Care Open. 2018;3(1):e000164. [27]	4 (29%)	6 (43%)	13	
Becker BA et al.: Emergency biliary sonography: utility of common bile duct measurement in the diagnosis of cholecystitis and choledocholithiasis. J Emerg Med. 2014;46(1):54–60. [28]	4 (29%)	5 (36%)	6	
Ralls PW et al.: Real-time sonography in suspected acute cholecystitis. Prospective evaluation of primary and secondary signs. Radiology. 1985;155(3):767–71. [29]	3 (21%)	2 (14%)	5	
Villar J et al.: The absence of gallstones on point-of-care ultrasound rules out acute cholecystitis. J Emerg Med. 2015;49(4):475–80. [30]	3 (21%)	1 (7%)	5	
Jang TB et al.: The learning curve of resident physicians using emergency ultrasonography for cholelithiasis and cholecystitis. Acad Emerg Med. 2010;17(11):1247–52. [31]	4 (29%)	1 (7%)	3	

## Discussion

Summaries of the top five papers for renal POCUS and biliary POCUS are provided below.

Renal POCUS

1. Smith-Bindman R et al.: Ultrasonography versus computed tomography for suspected nephrolithiasis. N Engl J Med. 2014;371(12):1100-10. [11]

In this landmark study, the authors compared computed tomography (CT), radiology ultrasound (RUS), and emergency physician (EP) POCUS in patients presenting to the emergency department (ED) with suspected nephrolithiasis. Adult patients were enrolled across 15 academic EDs and prospectively randomized to one of these three imaging arms. There were three primary outcomes: high-risk diagnoses with complications related to missed or delayed diagnosis, cumulative radiation exposure, and total costs. The investigators randomized 2759 patients in the intention-to-treat population, with 908 in the POCUS arm, 893 in the RUS arm, and 958 in the CT arm. A total of 113 patients were lost to follow-up. There was no difference in demographics, pain scores, medical history, or EP assessment of the likelihood of an alternate diagnosis between the study arms. There was no difference in serious adverse events among the study groups. Furthermore, there was no difference in high-risk diagnoses with complications during the first 30 days between study groups; only a total of 11 patients had this outcome. Diagnostic accuracy for nephrolithiasis demonstrated that ultrasound had lower sensitivity and higher specificity than CT. POCUS had a sensitivity of 0.54 (95% confidence interval (CI) 0.48-0.60) and specificity of 0.71 (95% CI 0.67-0.75), RUS had a sensitivity of 0.57 (95% CI 0.51-0.64) and specificity 0.73 (95% CI 0.69-0.77), and CT had a sensitivity of 0.88 (95% CI 0.84-0.92) and specificity of 0.58 (95% CI 0.55-0.62). In patients undergoing a single imaging examination, the ED length of stay was shorter in the POCUS group by 1.3 hours. This large study demonstrates the safety of POCUS as the initial imaging modality in patients with suspected nephrolithiasis, resulting in a shorter ED length of stay and decreased radiation exposure with a safety profile comparable to radiology-performed imaging.

2. Wong C et al. The accuracy and prognostic value of point-of-care ultrasound for nephrolithiasis in the emergency department: a systematic review and meta-analysis. Acad Emerg Med. 2018;25(6):684-98. [12]

This high-quality systematic review evaluated both the accuracy of POCUS for the diagnosis of nephrolithiasis and the prognostic value of POCUS in the management of renal colic. The authors included studies of adult patients presenting to the ED with renal colic symptoms. POCUS was considered positive when it identified any degree of hydronephrosis and when accepted criterion standards included CT evidence of renal stone or hydronephrosis, direct stone visualization, or surgical findings. Five studies with 1,773 patients were included in the meta-analysis for diagnostic accuracy. For all stones, POCUS had modest accuracy with a sensitivity of 0.70 (95% CI 0.67-0.73), specificity of 0.75 (95% CI 0.73-0.78), a positive likelihood ratio (+LR) of 2.85, and a negative LR (-LR) of 0.39. POCUS for renal colic performed markedly better when moderate or greater hydronephrosis was identified. The sensitivity, specificity, +LR, and -LR in this scenario were 0.29 (95% CI 0.25-0.32), 0.94 (95% CI 0.92-0.96), 5.22, and 0.76. Therefore, moderate or greater hydronephrosis detected by POCUS is clinically relevant as it is highly specific for nephrolithiasis and associated with stones >5 mm, which are more likely to require urological intervention.

3. Gaspari RJ, Horst K.: Emergency ultrasound and urinalysis in the evaluation of flank pain. Acad Emerg Med. 2005;12(12):1180-4. [13]

This prospective observational study evaluated the sensitivity and specificity of emergency POCUS for the diagnosis of renal colic. It was conducted in the ED of a large suburban teaching hospital. Patients were evaluated with renal POCUS to specifically identify hydronephrosis. Every patient had the same workup, including urinalysis, POCUS, CT, and a follow-up interview at one month. The POCUS assessment was performed by six EPs who had completed a minimum of 25 renal scans prior to participation in the study. The criterion standard for renal colic was based on one of the following: CT interpretation by the attending radiology physician demonstrating a kidney stone or passage of a kidney stone; passage of a kidney stone in a urine strainer within one month; or surgical intervention for a kidney stone within one month. The authors enrolled a convenience sample of 104 adult patients presenting with flank pain. Renal colic was diagnosed in 62 patients at one-month follow-up; of these, 58 were diagnosed by CT. While hematuria on urinalysis had high sensitivity for nephrolithiasis (0.93 (95% CI 0.87-0.97)), it had low specificity (0.33 (95% CI 0.25-0.39)). POCUS had high accuracy for renal colic when compared to the attending radiology physician's interpretation of the CT, with a sensitivity of 0.83 (95% CI 0.73-0.88) and specificity of 0.92 (95% CI 0.80-0.98). The addition of renal POCUS significantly improves diagnostic accuracy in patients with suspected renal colic.

4. Wang RC et al.: Effect of an ultrasound-first clinical decision tool in emergency department patients with suspected nephrolithiasis: a randomized trial. Am J Emerg Med. 2022;60:164-70. [14]

This pragmatic randomized trial assessed the impact of an electronic medical record (EMR)-integrated clinical decision support (CDS) aid on CT utilization, radiation dose, and ED revisits. It was performed in the ED of a single academic center. Providers ordering a CT scan for suspected nephrolithiasis were randomized to either usual care or the CDS intervention. In the CDS intervention, when providers ordered a CT, they were prompted to complete a CDS tool suggesting the provider either perform renal POCUS or order RUS instead in appropriate low-risk patients. Over a one-year period, 254 patients who had a CT scan ordered for nephrolithiasis were enrolled and randomized to either the usual care group or the CDS intervention group in a 1:1 ratio. There was a significant difference in CT completion between the groups: 87% (111/128) in the CDS group compared to 94% (119/126) in the control group, with a risk difference of -7.7% (95% CI -14.8 to -0.6%). There was no difference between the groups in total radiation exposure, ED revisits, or hospitalizations at seven or 30 days. An ultrasound-first CDS tool can reduce CT use in low-risk nephrolithiasis patients without increasing ED revisits or hospitalizations.

5. Taylor M et al.: Ultrasonography for the prediction of urological surgical intervention in patients with renal colic. Emerg Med J. 2016;33(2):118-23. [15]

This ED-based study attempted to determine the findings on RUS that predict surgical intervention. The authors performed a retrospective cohort analysis of 500 consecutive adult patients with a diagnosis of renal colic who underwent RUS during their ED visit at two academic tertiary care departments. Charts were reviewed for patient characteristics, diagnostic test results, and the need for surgical intervention (defined as extracorporeal shock wave lithotripsy, percutaneous nephrostomy, or ureteroscopy) within 16 weeks of the index visit. Of this cohort, 483 met eligibility criteria, and 349 had stones identified, with 162 stones measured to be ≥6 mm. Mild, moderate, and severe hydronephrosis were identified in 184 (38%), 72 (15%), and 3 (0.6%) patients, respectively. In the follow-up period, 67 patients had urologic surgical intervention. The presence of either a stone or moderate to severe hydronephrosis on ultrasound had a sensitivity of 0.97 (95% CI 0.89-1.00) in diagnosing the need for surgical intervention, but only a specificity of 0.28 (95% CI 0.24-0.33). Alternatively, the presence of both a stone and moderate to severe hydronephrosis on ultrasound had a specificity of 0.91 (95% CI 0.88-0.94) but only a sensitivity of 0.34 (95% CI 0.23-0.47). The clinical significance of these sonographic findings is important given their predictive value for surgical intervention.

Biliary POCUS

1. Ross M et al.: Emergency physician-performed ultrasound to diagnose cholelithiasis: a systematic review. Acad Emerg Med. 2011;18(3):227-35. [20]

This methodologically rigorous systematic review evaluated the diagnostic accuracy of EP-performed POCUS for cholelithiasis in ED patients presenting with symptoms consistent with biliary colic. The search ultimately yielded eight studies meeting inclusion criteria, with a total of 710 patients who underwent both EP-performed POCUS and a criterion reference standard. Acceptable reference standards included RUS, CT, magnetic resonance imaging (MRI), or surgical findings. The prevalence of gallstones in this population ranged from 46% to 80%, with a median prevalence of 60%. There was no implicit variation in accuracy across studies due to operator-dependent differences. The pooled sensitivity and specificity were 0.90 (95% CI 0.86-0.93) and 0.88 (95% CI 0.84-0.91), respectively, and the +LR was 7.5 and the -LR 0.12. By confirming that the accuracy of POCUS for cholelithiasis was similar to that reported for RUS, this systematic review provided a sound basis to justify the routine adoption of biliary POCUS for cholelithiasis in EM.

2. Hilsden R et al.: Point of care biliary ultrasound in the emergency department (BUSED) predicts final surgical management decisions. Trauma Surg Acute Care Open. 2022;7(1):e000944. [21]

This prospective cohort study assessed the utility of POCUS compared to RUS in guiding the clinical management decisions of surgeons treating biliary disease. ED patients ≥18 years of age presenting with abdominal pain to a single Canadian tertiary care centre were eligible for enrollment if the EP felt the clinical and POCUS findings were consistent with biliary disease. Patients were excluded if they underwent emergency surgery before RUS or were unable to consent. Of note, the POCUS images were recorded in a middleware system and published to the electronic medical record. After EP assessment, enrolled patients were referred to the general surgeon on call, and the surgical consultant was asked to make and record a clinical decision based on the history, physical exam, available lab work, and the POCUS scan without knowledge of any radiology imaging results. All patients then had RUS ordered and performed; they were followed through their hospital course, and their actual management served as the comparator to the POCUS-based management decision that had previously been recorded. Clinical management decisions were categorized into one of three options: surgery alone, duct clearance, or no surgery. Of the 103 patients enrolled, 100 were included in the final analysis. For the primary outcome comparing clinical management based on POCUS vs. RUS, the addition of RUS did not change management in 90% of patients. These results support the utility of POCUS to reliably inform surgical decision-making in the absence of radiology imaging in uncomplicated cases of biliary disease.

3. Summers SM et al.: A prospective evaluation of emergency department bedside ultrasonography for the detection of acute cholecystitis. Ann Emerg Med. 2010;56(2):114-22. [22]

This prospective observational study of ED patients presenting with suspected cholecystitis sought to determine the test characteristics of EP-performed POCUS and RUS for the diagnosis of cholecystitis as defined by a criterion standard of surgical pathology. The authors enrolled a convenience sample of patients ≥18 years of age with right upper quadrant pain, epigastric pain, vomiting, or fever. Patients were excluded if they were lost to follow-up or if no surgical pathology report was available. They enrolled 193 patients, of whom 189 were evaluated with biliary POCUS by the treating EP. Of this group, 25 patients were excluded: 23 were lost to follow-up and two had unavailable pathology reports, leaving 164 patients for final data analysis. The scans were performed by 43 EM residents and attending physicians, with each EP performing a median of two scans. POCUS had a sensitivity of 0.87 (95% CI 0.66-0.97) and a specificity of 0.82 (95% CI 0.74-0.88) for cholecystitis. This was similar to the diagnostic accuracy of RUS in this cohort, with a sensitivity of 0.83 (95% CI 0.61-0.95) and specificity of 0.86 (95% CI 0.77-0.92). Pericholecystic fluid was the least sensitive finding (0.26) but was the most specific (0.94). Within a two-week follow-up period, none of the patients who were discharged home after only a POCUS scan had a cholecystectomy or admission for cholecystitis. In conclusion, POCUS demonstrates good test characteristics for diagnosing cholecystitis.

4. Blaivas M et al.: Decreasing length of stay with emergency ultrasound examination of the gallbladder. Acad Emerg Med. 1999;6(10):1020-3. [23]

This retrospective study investigated if patients undergoing EP-performed biliary POCUS would have a different ED length of stay (LOS) compared to patients undergoing only RUS. The authors performed a retrospective chart review over a three-year period at their high-acuity, urban community hospital ED, which had an EM residency program. They identified 1,242 patients presenting with complaints consistent with gallbladder disease who underwent ultrasound assessment of the gallbladder (either POCUS, RUS, or both). Patients who were assessed with both POCUS and RUS were categorized into the POCUS group. The POCUS group had 753 patients, of whom 258 had further biliary RUS, and the RUS group had 489 patients. The POCUS group had a shorter ED length of stay (median 5 hr 15 min) compared to the RUS group (median 5 hr 37 min), with a median difference of 22 min (p=0.017, 95% CI 4-41 min). The largest effect was seen in patients presenting to the ED between 6 p.m. and 6 a.m. who were discharged, with a median 1 hr 3 min shorter LOS in the POCUS group. Sonographer experience also influenced LOS, with more experienced operators who had performed over 100 scans delivering shorter ED LOS compared to less experienced users. This study demonstrated that in an ED with an EM residency program and experience with biliary POCUS, there was an association between EP-performed POCUS and decreased ED LOS for patients presenting with biliary complaints, especially after hours.

5. Kendall JL, Shimp RJ.: Performance and interpretation of focused right upper quadrant ultrasound by emergency physicians. J Emerg Med. 2001;21(1):7-13. [24]

This prospective study enrolled a convenience sample of patients between 1996 and 1997 who underwent both right upper quadrant ultrasound (RUS) for abdominal pain or jaundice and EP-performed POCUS. This makes it one of the earliest studies exploring the diagnostic test characteristics of EP-performed biliary POCUS. All enrolling physicians completed a total of 11 lecture hours on POCUS for a variety of applications (including three hours on abdominal ultrasound) as well as 10 hours of hands-on training prior to enrolling patients in the study. Of the 112 patients enrolled, a total of 109 patients were included in the final analysis, of whom 51 had gallstones. EPs were found to be efficient with their POCUS scans, with 83% completed in less than 10 minutes. EP-performed POCUS had a sensitivity of 0.96 (95% CI 0.87-0.99) and specificity of 0.88 (95% CI 0.77-0.95) for gallstones when compared to RUS as the reference standard. Beyond completion of the prerequisite ultrasound training, physician experience with focused right upper quadrant ultrasound was not observed to be a major predictor of diagnostic accuracy. Notably, when compared to the final pathology report, the EP-performed sonographic Murphy sign was more sensitive than the sonographer-performed Murphy sign (0.75 vs. 0.45) for acute cholecystitis, underscoring the value of physician-patient contact during the scan.

Limitations

Because our panel consisted exclusively of Canadian EM POCUS experts, experts from different specialties outside of EM, like urology, general surgery, and radiology, may have selected different papers. Of note, one of the renal papers [15] did not actually study POCUS but focused on RUS performed on ED patients. However, their results demonstrating that sonographic findings are predictive of urological intervention are relevant to the use of POCUS in patients presenting with renal colic. As this was a modified Delphi process, we did not perform a systematic review or generate an exhaustive list of published papers on renal and biliary POCUS. Our methodology had the constraint of generating a top-five list; there are clearly additional papers of value in tables one and two that POCUS-engaged clinicians should be familiar with. Due to the system of weighted points based on individual rank lists, one of the biliary papers [24] with a low consensus based on the total number of votes made it into the top five list ahead of three other papers [25-27] with a higher consensus based on the total number of votes. Some members of the expert panel were authors of some of the candidate papers, and it is possible this may have biased the way they voted and created their rank lists in the three rounds of the modified Delphi process. This effect was likely minimized given the panel consisted of 14 members.

## Conclusions

This list of the top five influential papers for renal POCUS and biliary POCUS will be a useful resource for residents, fellows, and clinicians who want to support their clinical use of POCUS with the current literature base. While it is specifically relevant to EM practitioners, this list should also be informative to clinicians of all specialties who want to apply renal and biliary POCUS in an evidence-based manner. Future iterations of this process will continue to generate lists of the most influential POCUS papers published for other important applications and uses of POCUS.

## Appendices

Appendix One 

The names of the 14-member expert panel from the CAEP Emergency Ultrasound Committee and the Canadian Ultrasound Fellowship Collaborative.

**Table 3:** Expert panel members listed in alphabetical order and their academic affiliations.

Expert panel member	Academic university affiliation
Paul Atkinson	Dalhousie University
Colin Bell	University of Calgary
Talia Burwash-Brennan	Université de Montréal
Ian Buchanan	McMaster University
Jordan Chenkin	University of Toronto
Claire Heslop	University of Toronto
Tom Jelic	University of Manitoba
Daniel Kim	University of British Columbia
Elizabeth Lalande	Université Laval
David Lewis	Dalhousie University
Frank Myslik	Western University
Paul Olszynski	University of Saskatchewan
Gillian Sheppard	Memorial University of Newfoundland
Rajiv Thavanathan	University of Ottawa
